# Local Dominant Directional Symmetrical Coding Patterns for Facial Expression Recognition

**DOI:** 10.1155/2019/3587036

**Published:** 2019-05-13

**Authors:** Ying Tong, Rui Chen

**Affiliations:** College of Communication Engineering, Nanjing Institute of Technology, Nanjing 211167, China

## Abstract

To overcome the shortcomings of inaccurate textural direction representation and high-computational complexity of Local Binary Patterns (LBPs), we propose a novel feature descriptor named as Local Dominant Directional Symmetrical Coding Patterns (LDDSCPs). Inspired by the directional sensitivity of human visual system, we partition eight convolution masks into two symmetrical groups according to their directions and adopt these two groups to compute the convolution values of each pixel. Then, we encode the dominant direction information of facial expression texture by comparing each pixel's convolution values with the average strength of its belonging group and obtain LDDSCP-1 and LDDSCP-2 codes, respectively. At last, in view of the symmetry of two groups of direction masks, we stack these corresponding histograms of LDDSCP-1 and LDDSCP-2 codes into the ultimate LDDSCP feature vector which has effects on the more precise facial feature description and the lower computational complexity. Experimental results on the JAFFE and Cohn-Kanade databases demonstrate that the proposed LDDSCP feature descriptor compared with LBP, Gabor, and other traditional operators achieves superior performance in recognition rate and computational complexity. Furthermore, it is also no less inferior to some state-of-the-art local descriptors like as LDP, LDNP, es-LBP, and GDP.

## 1. Introduction

Facial expressions are one of the most important human body language constituents, which can accurately express personal emotions and mental and psychological conditions. In recent years, computer analysis has been used to better understand human facial expressions and has presented an important prospect of application in human-machine interaction, which has drawn a lot of attention [1–4]. For example, video cameras have become an integral part of many consumer devices and can be used for capturing facial images for recognizing people and their emotions. Generally, facial expression feature extraction and classification are the two most critical steps for accurate facial expression recognition (FER) system. Considering that the description precision of different expression features will directly affect the performance of classification, in this paper, we focus on finding a fast and efficient facial expression feature descriptor.

In many facial feature extraction methods, local feature descriptors have strong robustness to the influence of illumination, noise, and other interference. Local Binary Patterns (LBPs) [5] and Gabor transformation [6–8] are two most representative methods. Gabor transformation could extract multiscale, multidirectional texture information of images, but its feature size is prohibitively large which results in higher computational complexity. Therefore, it would be desirable to adopt a suitable feature selection method (or dimensionality reduction method) to reduce feature size but still maintain superior recognition performance. Compared with Gabor transformation, LBP descriptor has the advantages of simple theory and easy implementation which has been extensively used in face classification, image retrieval, and object tracking [9–12]. However, the traditional LBP algorithm only encodes the gray-value differences between each central pixel and its eight neighbourhood pixels and could not precisely represent the structural and directional information of neighbourhood pixels which is extremely significant for facial feature description. Researchers have proposed some improved LBP-based algorithm which achieved good performance, but they are inevitably at the cost of expensively computational complexity [13–16].

**Figure 1:**
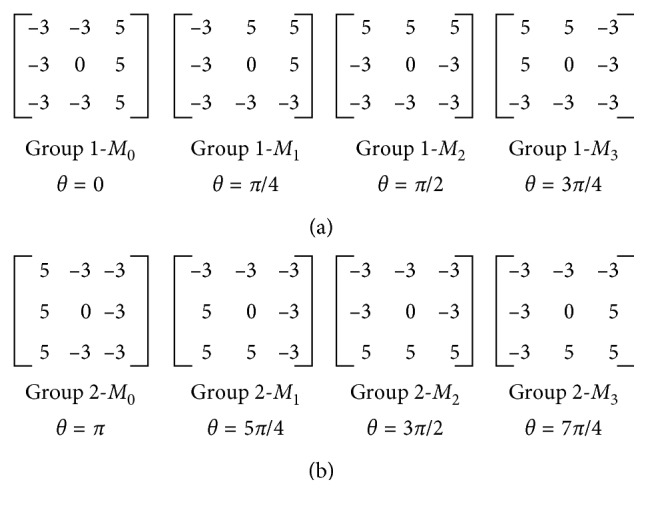
Two groups of direction masks. (a) The first group. (b) The second group.

**Figure 2:**
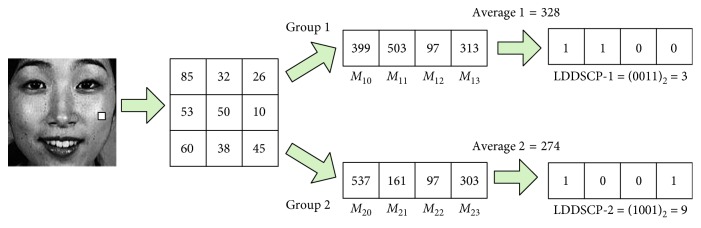
The coding sketch of LDDSCP encoder. Two groups of symmetrical direction masks are convoluted with the original expression image, and the corresponding convolution values of each group are presented in the middle of sketch. Then, we encode the dominant direction information by comparing each convolution value with the average strength of its belonging group and obtain the LDDSCP-1 code and LDDSCP-2 code subsequently (shown in the right).

In view of this, we propose a novel local feature descriptor named as Local Dominant Directional Symmetrical Coding Patterns (LDDSCPs) which not only possesses superior performance but also has lower computational cost at the same time. (1) Inspired by human visual characteristics, namely, being more sensitive to directional information than contrast information and colour information of objects, LDDSCP encodes the dominant direction information of facial expression textures based on two groups of symmetrical direction masks as shown in Figure 1. The coding procedure of LDDSCP descriptor is illustrated in Figure 2. In detail, we firstly adopt each direction mask to convolve facial expression images. Then, the obtained convolution value based on direction mask is compared to the average strength of its belonging group for getting two groups of binary codes denoted as LDDSCP-1 code and LDDSCP-2 code, respectively. Since the weight distribution in each direction mask successively shows different direction information, the strength difference between four convolution values in each group can characterize the intensity variations of eight neighbourhood pixels in the corresponding directions. Therefore, LDDSCP encoder can more precisely characterize the prominent direction and structure of facial expression textures hidden in the deformation of eyes, mouth, and other facial areas. (2) Due to the symmetry of two groups of direction masks, the obtained codes of LDDSCP-1, and LDDSCP-2 present the complementary information each other. The final LDDSCP feature vector is formed by stacking the histograms of LDDSCP-1 codes and LDDSCP-2 codes, which not only compensates for the loss of direction information in the LDDSCP-1 codes or LDDSCP-2 codes, but also reduces the feature dimension and computational complexity. Experimental results on the JAFFE and CK databases show that the proposed LDDSCP descriptor consistently achieves superior performance compared with some traditional descriptors like as LBP [17], CS-LBP [18], Gabor [19], and HOG [20] and outperforms four state-of-the-art descriptors, e.g., LDP [13], LDNP [14], es-LBP [15], and GDP [21], from two aspects of recognition accuracy and running time.

The remainder of this paper is organized as follows. In Section 2, we introduce the coding scheme of LDDSCP and analyse its robustness. In Section 3, we present the implementation steps of LDDSCP feature descriptor.  Section 4 provides and discusses the experimental results on the JAFFE database and CK database, and Section 5 is the conclusion of our paper.

## 2. Local Dominant Directional Symmetrical Coding Patterns

### 2.1. Difference with Previous Works

Ojala et al. [5, 22] firstly proposed Local Binary Pattern and its uniform pattern which have been widely used in local feature extraction for face image analysis in recent years. After researching and analysing, we found that it has two crucial shortcomings. One is that LBP descriptor only encodes the gray-value differences between each central pixel and its neighbourhood pixels into an 8-bit binary code, which neglects most intensity variations among neighbourhood pixels and is sensitive to the influence of environmental illumination, random noise, and other inference. Another is that the feature size of LBP is in proportion to bit numbers of binary code and block numbers of image division, which causes the expensive computational cost. In view of this, many improvements based on LBP feature descriptors were put forward by researchers. Huang et al. [23, 24] proposed extended LBP to encode the gray-value differences between the central pixel and its neighbors into a 4-bit binary code, of which the first bit presented the sign of gray-value difference and the rest three bits encoded the absolute value of gray-value difference. Local Ternary Pattern (LTP) [25] was proposed by encoding the relationships between gray-value differences and their threshold value, which improves the robustness of noise and illumination. Expression-specific LBP (es-LBP) [15] was presented by emphasizing the partial information of human faces on particular fiducial points.

Even though these feature descriptors make some improvements aiming at the shortcomings of LBP algorithm, their basic coding schemes are monotonous, namely, only encoding the gray-value differences between each central pixel and its neighbors without considering the intensity variations among neighbourhood pixels. In fact, the intensity variations imply more abundant directional information of textural structure that is more benefit to the representation of facial expression features. Hence, some notable feature descriptors [13, 14, 16, 26–32] were proposed subsequently. Jabid et al. [13, 14, 29] proposed LDP, LDPv, and LDNP descriptors, respectively, for facial expression recognition by encoding the directional information of facial textures. Lou et al. [30] provided Local Line Directional Patterns (LLDPs) in which the modified finite radon transform (MFRAT) and the real part of Gabor filters are exploited simultaneously. Yuan [31] presented a high-order Derivation Local Binary Pattern (DLBP) by encoding the sequential binary values of high-order directional derivatives. Furthermore, for improving the discriminative capability, circular shift subuniform patterns and scale space theory are applied in DLBP for obtaining scale and rotation invariance. Mu et al. [32] proposed a shift and gray scale invariant feature descriptor by using shiftable complex directional filter bank (CDFB) transform and uniform LBP encoder. More recently, Patterns of Oriented Edge Magnitudes (POEMs) [26], Dual-Cross Patterns (DCPs) [16], three-patch LBP [27], and four-patch LBP [28] have also been presented for enhancing the precision of feature representation.

In summary, these above descriptors make improvements on the traditional coding scheme of LBP and have superior performance on facial expression recognition, face recognition, texture classification, and palmprint identification. However, compared with our proposed method, there are still some differences. We make the following observations:

1. Although LDP [13], LDPv [29], and LDNP [14] are somewhat similar to our proposed method, compared with them, the proposed LDDSCP descriptor has two different contributions. One is that LDDSCP encoder has the characteristics of symmetry and direction which are in accordance with human visual characteristics. Another is that LDDSCP descriptor stacks the LDDSCP-1 histogram and the LDDSCP-2 histogram which not only increases the performance but also reduces the computational cost.
2. As depicted in References [30, 31], LLDP [30] encodes the structure of a local neighborhood by calculating the sum of gray values of pixels in a line direction, and DLBP [31] encodes the high-order derivative in different directions. Compared with our proposed LDDSCP encoder, they only consider the gray-value differences between two pixels or the intensity sum of a series of pixels and ignore the overall gray-value distribution among neighbourhood pixels. Hence, compared to our proposed LDDSCP descriptor, LLDP and DLBP are more sensitive to the influence of environmental illumination and noise inference.
3. Analysing the procedures of LLDP [30], DLBP [31], and the method [32] presented by Mu et al., we find that these methods all exploit assistant ways to further improve the discriminative performance. For example, LLDP [30] adopted the modified finite radon transform (MFRAT) and the real part of Gabor filters simultaneously for getting the precise line-geometry features. DLBP [31] introduced circular shift subuniform patterns and scale space theory for obtaining scale and rotation invariance. Mu et al. used shiftable complex directional filter bank (CDFB) transform to capture the energy shiftable information. Thus, it can be seen that the additional computational expense of these descriptors is exchanged for high performance. This strategy has not been adopted in our proposed method.
4. Although POEM [26], DCP [16], three-patch LBP [27], and four-patch LBP [28] still have superior performance without adopting specific filters to process images, their coding schemes are tedious and complicated, which inevitably results in higher computational complexity and restricts the practical applications. In comparison with them, LDDSCP descriptor has concise theory and easy implementation.

### 2.2. Implementation Ideas of LDDSCP Feature Descriptor

**Figure 3:**
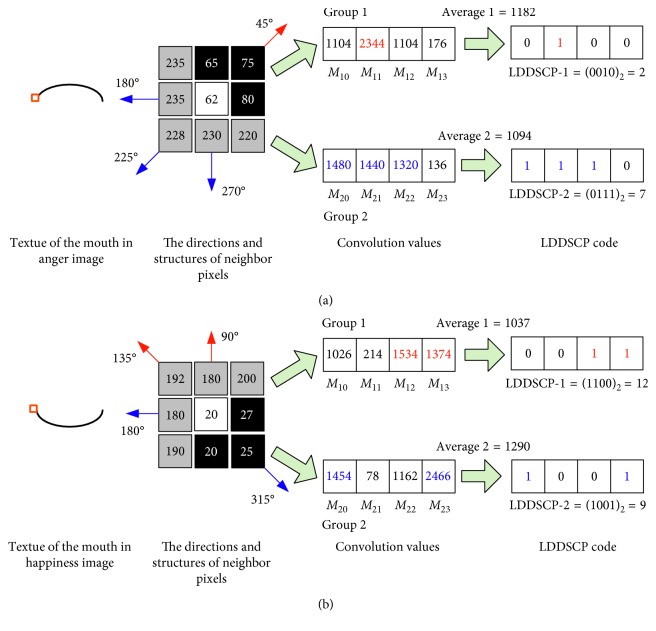
Comparison of LDDSCP-1 code and LDDSCP-2 code in two different expression textures.

**Figure 4:**
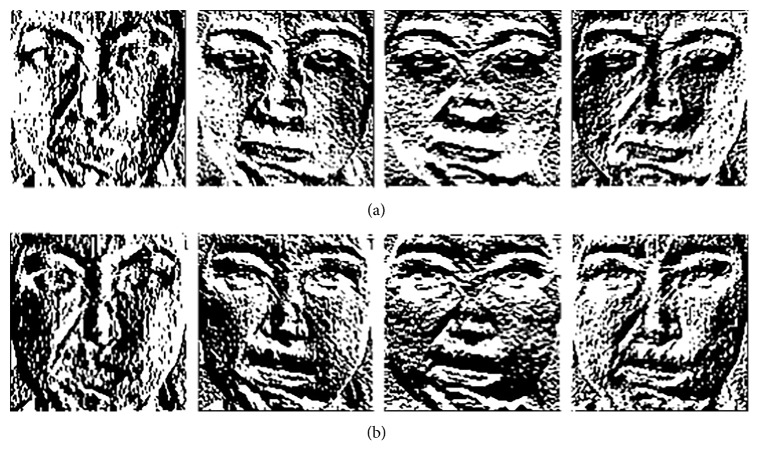
Convolution images of an example image based on two groups of symmetrical direction masks. (a) The convolution images of the first group and (b) the convolution images of the second group.

To avoid this problem, in this paper, we present LDDSCP feature descriptor which not only precisely capture facial expression features but also greatly reduces computational costs. The pivotal implementation ideas are as follows:

1. In view of the sensitive directions of human eyes only ranging between 0° and 180° [33, 34], we divide eight convolution masks into two symmetrical groups according to their directions. Each group includes four masks as shown in Figure 1, and the direction of each mask is defined in the light of its weight distributions. It is obvious that the weight distributions of each mask in the first group successively appears in the directions of 0°, 45°, 90°, and 135° and the second group's weight distributions are manifested in the directions of 180°, 225°, 270°, and 315° in sequence which are symmetrical with the directions of the corresponding masks in the first group. Due to the range of the directions in each group being not more than 180°, the proposed grouping idea is a feasible mechanism which conforms to human visual characteristics.
2. Considering that human visual system is more sensitive to directional information than contrast information or tinctorial information, we calculate the different convolution values of each pixel based on two groups of symmetrical direction masks and compare each convolution value with the average strength of its belonging group to encode the dominant direction information of facial expression textures. The elaborate analysis process is illustrated in Figure 3. There are two different kinds of texture examples which could concisely depict the mouth deformation in anger images and happiness images, and the neighbor's gray-values of the sampled pixel (marked by orange box in two expression textures) are also presented (the neighbors of anger texture are located in the left-top, and the neighbors of happiness texture are in the left-bottom). It is observed that the structures of eight neighbors in two kinds of expression textures are all composed of black part (lower gray-values) and hoar part (higher gray-values), but the dominant directions revealed by texture structures are entirely different. In details, the structure of anger texture is appeared in the directions of 45°, 180°, 225°, and 270°, while the structure of happiness texture is manifested in the directions of 90°, 135°, 180°, and 315°, which are not completely differentiated by LBP encoder (these two texture examples are all coded as “11111111”) but can be precisely represented by the LDDSCP encoder. That is the binary codes of LDDSCP-1 and LDDSCP-2 in the anger texture are “0010” and “0111”, respectively, while the binary codes of LDDSCP-1 and LDDSCP-2 in the happiness texture are “1100” and “1001”, of which “1” (red colour) of LDDSCP-1 code presents the dominant directions of textures at the range of 0° to 180°, and similarly, “1” (blue colour) of LDDSCP-2 code denotes the dominant directions of textures at the range of 180° to 360°. Apparently as shown in Figure 3, the red “1” of LDDSCP-1 code is consistent with the directions of red arrows, and the blue “1” of LDDSCP-2 code is also in line with the directions of blue arrows. Therefore, LDDSCP encoder can more accurately characterize the difference of dominant direction distributions between two different expression textures even though composed of the same structure composition, which is in accordance with human visual characteristics.
3. Due to the symmetry of direction masks of two groups, the resulting convolution images of two direction masks with a 180° difference show the same textural structures but are depicted by two opposite gray-values as shown in Figure 4. Hence, the expression information implied in the first group of convolution images is similar to the second group, and so the statistical histograms of LDDSCP-1 codes and LDDSCP-2 codes can be stacked into the final LDDSCP feature vector, which not only contains abundant facial expression information but also reduces the computational complexity.

In summary, even though the implementation ideas of LDDSCP descriptor are brief and uncomplicated, LDDSCP descriptor is provided with three outstanding advantages: (1) LDDSCP descriptor adopts the information of entire neighbourhood pixels, instead of the difference values between the central pixel and its neighbors for coding; (2) the coding scheme is on the basis of the dominant direction information of each group, instead of the intensity values of each pixel, which has more robustness on the intensity change of facial expression images; and (3) the strategy using stacking histograms is an efficient fusion method of feature vector which not only retains a lower feature dimension but also possesses higher discriminative power.

### 2.3. Coding Scheme

Considering that Kirsch masks can more accurately detect different directional texture information than other convolution masks, we apply Kirsch masks into LDDSCP coding scheme. As depicted in previous sections, we encode the dominant direction information of facial expression texture in each group and obtain two groups of 4-bit binary codes named as LDDSCP-1 code and LDDSCP-2 code, respectively. These two binary codes in each pixel are represented as

(1) $$\begin{array}{c} \text{LDDSCP}-1=\overset{3}{\underset{i=0}{\sum }}S\left(M_{1i}-{\text{average}}_{1}\right)\times 2^{i},\\ {\text{average}}_{1}=\frac{1}{4}\overset{3}{\underset{i=0}{\sum }}M_{1i}, \end{array}$$

(2) $$\begin{array}{c} \text{LDDSCP}-2=\overset{3}{\underset{i=0}{\sum }}S\left(M_{2i}-{\text{average}}_{2}\right)\times 2^{i},\\ {\text{average}}_{2}=\frac{1}{4}\overset{3}{\underset{i=0}{\sum }}M_{2i}, \end{array}$$

where $S\left(x\right)=\left\{\begin{array}{cc} 1, & x\geq 0,\\ 0, & x<0, \end{array}\right.$ and *M*_*ji*_ is the convolution value of four directional masks (*i*=1,2,3,4) of two groups (*j*=1,2), and average_*j*_ is the mean value of four convolution values of the corresponding group. We encode the difference between *M*_*ji*_ and average_*j*_ to obtain a 4-bit binary code by LDDSCP-1 encoder or LDDSCP-2 encoder, where “1” indicates the prominent texture deformation of facial expression in some directions and conversely “0” represents the indistinctive texture deformation in the remaining directions. Therefore, each of the 4-bit binary code defines one kind of local texture pattern except “0000” and “1111”, which can be more insensitive to illumination, noise, and other inference. Because “0000” and “1111” all have no actual meaning on describing the dominant direction information of texture structures, the total number of LDDSCP-1 codes or LDDSCP-2 codes is ultimately equal to 2^4^ − 2 = 14. Hence, the number of histogram bins per block of LDDSCP is only 14 by stacking strategy which is reduced by 94.53% than the LBP descriptor.

### 2.4. Robustness of LDDSCP Encoder

As introduced in Section 2.2, we have demonstrated that LDDSCP encoder can precisely characterize the textural direction information even if represented by the same structure composition. In this section, we further analyse the robustness of LDDSCP encoder compared with LBP encoder.

**Figure 5:**
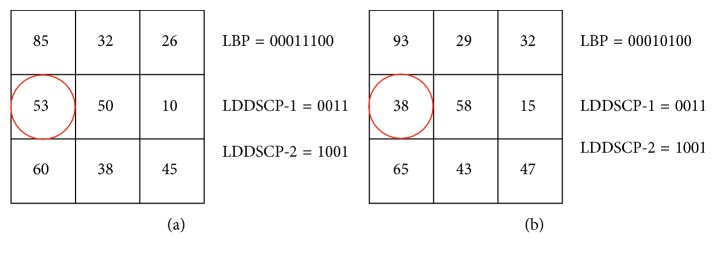
Robustness of LBP vs. LDDSCP. (a) The binary codes of LBP and LDDSCP in the original image patch; (b) the binary codes of LBP and LDDSCP in the noisy image patch.

Since LBP only encodes the difference value between the central pixel and neighbourhood pixels in the light of its theory, any change of logic relationship between these pixels results in a different LBP code. For instance, as shown in Figure 5, the binary code of LBP in the original image patch is “00011100” (as shown in Figure 5(a)); however, it is changed to “00010100” on account of noise inference (as shown in Figure 5(b)). In detail, the 4th bit of LBP code is only changed from “1” to “0” due to the reversed logic relationship between the central pixel and the fourth neighbourhood pixel circled by red round in Figure 5, while the remaining bits of LBP code are all still unchanged. Nevertheless, LDDSCP encoder provides the same coding patterns even if there is some presence of noise inference. Thus, it can be seen that the noise tolerance of LDDSCP encoder is greater than that of LBP encoder under the condition that the added noise is not enough to affect the expression textures.

## 3. LDDSCP Feature Descriptor

**Figure 6:**
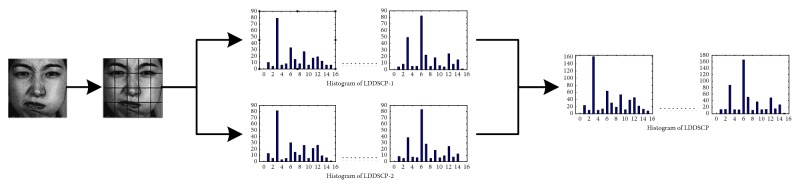
Schematic diagram of the proposed LDDSCP feature descriptor.

After encoding the input image by the LDDSCP encoder, two coded images are produced. In the case of classify, the coded image cannot be directly used as a feature vector. This is owing to that the code value of each pixel is closely relevant to its position in the image, and then the classification results based on the difference of code values will lead to greater errors on account of the inaccurate position offset. Furthermore, the feature vector composed by the coded images has a prohibitively large size which may unnecessarily increase the complexity of training and classification tasks. Hence, in this section, we still adopt the statistical histogram of LDDSCP descriptor as a feature vector to present the facial expression image. Implementation steps are detailed as follows:

(1) The original input image is partitioned into *N* *∗* *N* nonoverlapping blocks, and the size of each block is *m*  ×  *n* pixels.
(2) We, respectively, compute the decimal value of LDDSCP-1 code and LDDSCP-2 code by (1) and (2) in each pixel of each block.
(3) Two histograms of LDDSCP-1 code and LDDSCP-2 code are statistically obtained for each block:
(3) $$\begin{array}{c} H_{1}\left(i\right)=\overset{m}{\underset{r=1}{\sum }}\overset{n}{\underset{c=1}{\sum }}f\left(\text{LDDSCP}-1\left(r,c\right),i\right),\\ H_{2}\left(i\right)=\overset{m}{\underset{r=1}{\sum }}\overset{n}{\underset{c=1}{\sum }}f\left(\text{LDDSCP}-2\left(r,c\right),i\right), \end{array}$$
  where $f\left(x,y\right)=\left\{\begin{array}{cc} 1, & x=y,\\ 0, & x\neq y, \end{array}\right.$ and LDDSCP − 1(*r*, *c*) or LDDSCP − 2(*r*, *c*) are the decimal values of pixel (*r*, *c*), and (*i*(*i*=1,2,…, 2^*k*^ − 2)) is the traversed decimal values produced by LDDSCP-1 encoder or LDDSCP-2 encoder which has been detailed in the Section 2.3.
(4) Owing to that each block has two histograms, namely, *H*_1_ and *H*_2_, we, respectively, concatenate all *H*_1_ histograms of blocks and all *H*_2_ histograms of blocks in sequence to yield two histograms of image, namely, *H*_LDDSCP−1_ and *H*_LDDSCP−2_, which are represented as
(4) $$\begin{array}{c} H_{\text{LDDSCP}-1}=\overset{N*N}{\underset{k=1}{\prod }}H_{1}^{k},\\ H_{\text{LDDSCP}-2}=\overset{N*N}{\underset{k=1}{\prod }}H_{2}^{k}, \end{array}$$
  where ∏ is the concatenation operation and *N* × *N* is the number of blocks of the divided images. At last we stack the *H*_LDDSCP−1_ histogram and *H*_LDDSCP−2_ histogram to obtain the resultant LDDSCP histogram of image as the final feature vector. The schematic diagram of the proposed LDDSCP feature descriptor is shown in Figure 6.

## 4. Experiments

In the section, the proposed LDDSCP descriptor is evaluated and experiments are conducted on two well-known databases: JAFFE and Cohn-Kanade databases. The JAFFE database is provided by the ATR Media Information Science Laboratory in Japan, which contains 213 facial expression images of 10 Japanese females. These images are divided into 6 classes in the light of expression, namely, anger, disgust, fear, happiness, sadness, and surprise, and each subject per expression has three or more facial expression images. We chose 3 images of each expression per subject for getting a new subset totally including 180 expression images.

The Cohn-Kanade database is established by the Robotics Institute of Carnegie Mellon University, which consists of nearly 593 expression image sequences of 123 university students aged from 18 to 30 years old. Each image sequence of each subject is changed from the neutral expression to one of six target prototypic expressions. The size of images is 640 × 480 pixels or 640 × 690 pixels. We chose the five most representative image frames taken from 333 sequences, which resulted in 1665 expression images.

**Figure 7:**
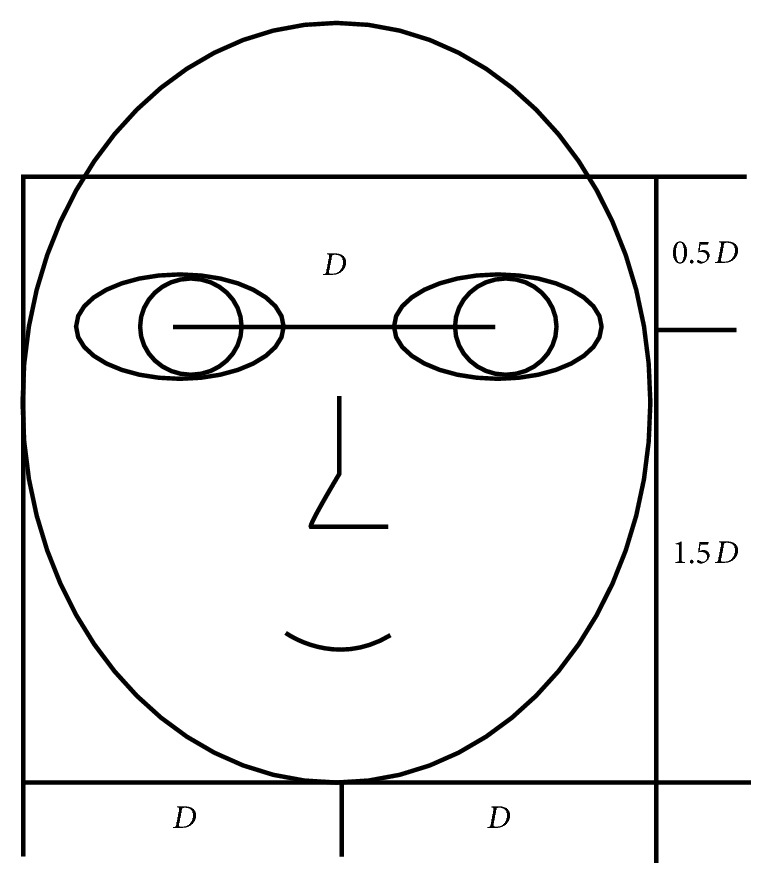
Cropping template of facial expression image.

**Figure 8:**
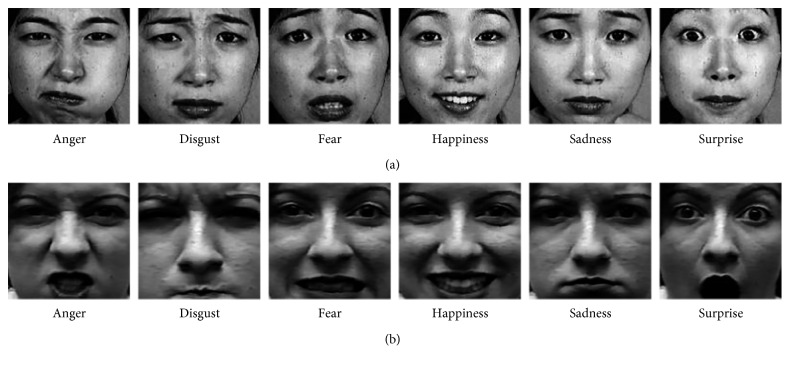
Example images of each prototypic expression from (a) JAFFE database and (b) CK database.

In our experiments, we firstly adopt a cropping template which is shown in Figure 7 to obtain the salient expression region which is resized to 128 × 128 pixels. The example images of each prototypic expression on two databases are shown in Figure 8. Then, we exploit KNN classifier and the “leave-one-sample-out” strategy (hereafter denoted as “L-O-Sam-O”) [7] to identify 6 classes of facial expression. The L-O-Sam-O strategy is an effective sample selection method. In each cross validation, one image is selected as the test sample, and the residual *N* − 1 images are selected as the training samples (*N* is the number of total samples, *N* = 180 for JAFFE database, and *N* = 1665 for CK database). *N* cross validations are carried out by traversing each sample to guarantee the precision of expression classification.

Four experiments are conducted. Firstly, we analyse the selection of optimal parameters aiming to the proposed LDDSCP descriptor. Secondly, two strategies of building histograms of LDDSCP descriptor are evaluated on both the computational complexity and the quality of the classification results. Thirdly, the performance of LDDSCP descriptor is compared with four traditional feature descriptors and three state-of-the-art feature descriptors. Finally, the robustness of LDDSCP is presented for recognizing two kinds of added noisy databases.

### 4.1. Optimal Parameters Selection of the LDDSCP Descriptor

Since the number of blocks into which the image is divided is the most important parameter that profoundly affects the accuracy of classification, we conduct four different cases: 2 × 2, 4 × 4, 8 × 8, and 16 × 16 blocks on the JAFFE database. The corresponding experimental results are shown in Table 1. It can be seen that the case of 8 × 8 blocks presents the optimum performance. At the case of 2 × 2 blocks, the LDDSCP descriptor could not extract abundant expression features resulting in lower recognition rate (73.89%). The recognition rate is increased with the number of blocks till the case of 8 × 8 blocks. That is because the greater the number of blocks is, the more valuable information the feature vector incorporates. However, after a certain point, too many blocks ought to result in producing unnecessarily redundant local information which can degrade the performance. Consequently, in the following experiments, we will consistently employ the optimal block parameter (8 × 8 blocks) on the performance evaluation.

### 4.2. Evaluation of Two Strategies of Building Histogram of the LDDSCP Descriptor

As introduced before, we achieved two statistical histograms of LDDSCP-1 and LDDSCP-2, respectively, and employed two strategies to get the final histogram of the LDDSCP descriptor. One is that concatenating the LDDSCP-1 histogram and the LDDSCP-2 histogram; another is that stacking the above two histograms. The experimental results of two strategies of building histogram of the LDDSCP descriptor are reported in Table 2. From it, we can see that the strategy of stacking histograms causes two advantages. (1) The strategy of stacking histograms can reduce the feature dimensions per block, and the computational cost is nearly half than the strategy of concatenating histograms, and (2) the strategy of stacking histograms compensates for the missed information of LDDSCP-1 histogram or LDDSCP-2 histogram in each other, and then the final histogram of LDDSCP descriptor contains abundant expression information which results in a higher recognition rate (94.44%) with a margin of 1.66% by comparing with the result of concatenating strategy. Thus, we adopt the strategy of stacking histograms in the following experiments on account of its aforementioned merits.

### 4.3. Performance Evaluation of the LDDSCP Descriptor

To better illustrate the advantages of LDDSCP, we compare it with four traditional descriptors, i.e., LBP [17], CS-LBP [18], Gabor [19], and HOG [20], as well as four state-of-the-art descriptors, i.e., LDP [13], LDNP [14], es-LBP [15], and GDP [21]. The default parameters of these above descriptors are set up by referring to their corresponding references and the optimum parameters of LDDSCP descriptor are selected according to the discussion in Section 4.1. Therefore, we list the essential parameters of each descriptor in Table 3.

In light of the above parameter settings, we conduct experiments on the JAFFE database and CK database, and the corresponding experimental results are shown in Tables 4 [Table tab5]–6, respectively. We make the following observations:

1. As depicted in Table 4, the feature size of LDDSCP is smaller than other descriptors, but its feature extraction time ranks third which is slightly longer than that of HOG and CS-LBP. This is because that the process of computing convolution values is more complicated than that of computing gradient values or difference values, it makes the increment of computational cost of LDDSCP feature extraction. However, in the step of classification, LDDSCP descriptor needs the least running time by reason of its smallest feature size. Consequently, LDDSCP descriptor possesses the least computation cost by combining feature extraction time with classification time.

where *d*_*i*_ is the distance between one sample and the center sample of each expression, and *s* is the number of expressions.

(2) Since the merit of LDDSCP descriptor is benefitted from its coding scheme, in this section, we only compare the performance of these feature descriptors which adopt the coding strategy (or frequency transformation) to obtain the final feature vectors without conjunction with additional methods for improving performance, such as filtering images, reducing dimensions, dividing fiducial regions, etc. The experimental results are more comparable and more persuasive as shown in Table 5. As it can be seen, excepted for Gabor descriptor which is based on frequency transformation, the remaining descriptors all lose to LDDSCP. In detail, LDDSP outperforms HOG by 3.88% on the JAFFE database and surpasses LDP by 0.36% on the CK database, respectively. Although the recognition rate of Gabor is slightly higher than that of LDDSCP on the JAFFE database, the computational cost of Gabor is far more than that of LDDSCP as shown in Table 4. Thereby, LDDSCP has the most superior performance by comprehensively considering recognition accuracy and computation time.

(3) For demonstrating the effectiveness of our coding scheme in this paper, we employ any group of direction masks to encode the dominant direction information of expression texture, and the obtained LDDSCP-1 feature vector and LDDSCP-2 feature vector are used for facial expression recognition, respectively. The experimental results are shown in Table 6. From it, we can see that LDDSCP-1 ranks second on the JAFFE database and LDDSCP-2 ranks second on the CK database. Furthermore, LDDSCP-1 and LDDSCP-2 are all superior to other feature descriptors, such as LBP, CS-LBP, LDP, etc. Thus, it proves that the scheme of encoding the dominant direction of facial textures is more conductive to capturing the rich and precise expression information compared with other feature descriptors.

(4) As shown in Table 5, the recognition rates of feature descriptors on JAFFE database are all relatively lower than those on CK database. In order to explain this phenomenon, we randomly select one fear image from the JAFFE database and CK database, respectively, and calculate the expression components:

(5) $$\begin{array}{c} \text{Ra}=\frac{1/{\left(d_{i}\right)}^{2}}{\sum_{i=1}^{s}1/{\left(d_{i}\right)}^{2}}. \end{array}$$

**Figure 9:**
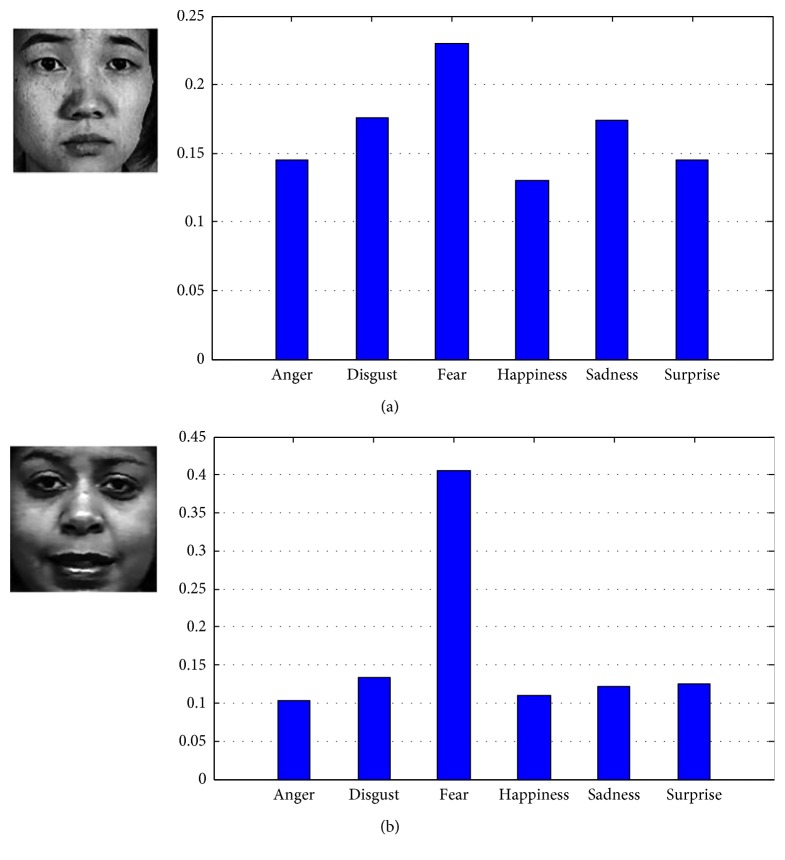
Expression components of the examples on different databases. (a) JAFFE database and (b) CK database.

The expression component values of two images are illustrated in Figure 9, respectively. In detail, six expression component values of one fear image on the JAFFE database are separately 14.51%, 17.58%, 22.95%, 13%, 17.44%, and 14.52%, while the corresponding values of one fear image on the CK database are 10.38%, 13.37%, 40.51%, 11.04%, 12.15% and 12.55%, respectively. As it can be seen, these six component values on the JAFFE database are approximate in which the fear component value is only slightly higher than other expression component values. Conversely, the component values on the CK database are discrepant where the fear component value is significantly higher than others especially. Thus, we draw a conclusion that the false identification possibilities of images labelled as the same expression are affected by the distribution of expression components, which has been verified in Table 5.

### 4.4. Robustness Evaluation of the LDDSCP Descriptor

**Figure 10:**
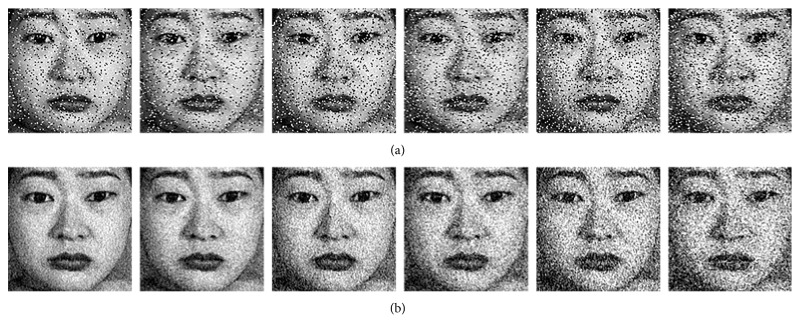
Some noise samples in which the intensity of noises is gradually increased. (a) Salt and pepper noise and (b) additive white Gaussian noise.

**Figure 11:**
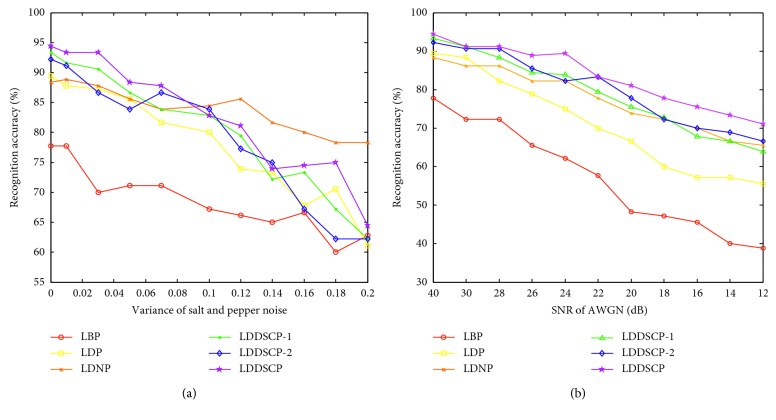
Recognition rates of different descriptors with different intensity of noises. (a) Salt and pepper noise and (b) additive white Gaussian noise.

Finally, we evaluate the noise-robustness of the LDDSCP descriptor by, respectively, adding two categories of noise on JAFFE database and using the KNN classifier. The SNR of additive white Gaussian noise (AWGN) is adjusted from 12 dB to 40 dB, and the variance of salt and pepper noise is modulated from 0 to 0.2. It should be noted that the noise image which is added AWGN with 40 dB SNR is equal to the original image, and the noise image which is added salt and pepper noise with 0 variance is also the original image. Some noise samples are depicted in Figure 10 where the intensity of noises is gradually increased, and the corresponding experimental results are shown in Figure 11. With the increment of noise intensity, the recognition rates of all descriptors are gradually decreasing, but the recognition rate of LDDSCP is higher than those of other descriptors under the overwhelming majority of noise conditions. Particularly in the analysis of average precision, LDDSCP outperforms LDP and LBP by 4.84% and 14.91% on the condition of salt and pepper noise and transcends LDP and LBP by 12.42% and 25.85% on the condition of AWGN. Thus, it can be seen that LDDSCP has a stronger noise-robustness. Additionally, the results of LDDSCP-1 or LDDSCP-2 as depicted in Figure 11 also further demonstrate that the combination of LDDSCP-1 and LDDSCP-2 is more beneficial to overcome the noise effectively.

## 5. Conclusions

Facial expression recognition is a challenging and cutting-edge technology. Due to the nonrigid character of human face, the facial expression is influenced by age, sex, hair, ethnicity, illumination, etc. Therefore, how to extract the precise expression features is the most critical step of facial expression recognition. For this goal, the work presented in this paper proposed the following contributions:

1. We introduced a novel coding scheme, LDDSCP, which encodes the dominant directions of facial textures effectively. Experimental results demonstrated that LDDSCP utilizes the dominant directional information which is more suitable to describe the expression feature of human face accurately.
2. Due to that human visual system is sensitive to directional information which is between 0 degree and 180 degree, we adopted the strategy of stacking histograms to obtain a compact feature. Experimental results proved that this strategy not only compensates for the missed information of LDDSCP-1 histogram or LDDSCP-2 histogram but also significantly reduces the computational cost.
3. At last, we also evaluated the performance of LDDSCP against some traditional descriptors and some state-of-the-art descriptors in noisy conditions. Detailed experiments showed that LDDSCP is also more stable against noise than intensity compared with other descriptors. Hence, LDDSCP has a stronger practicability on the basis of the above conclusions.

## Figures and Tables

**Table 1:** Recognition performance for different number of blocks (%).

Block numbers	2 × 2	4 × 4	8 × 8	16 × 16
Recognition rate	73.89	87.78	**94.44**	89.44

**Table 2:** Evaluation of two strategies of building histogram of the LDDSCP descriptor.

Strategy of building histogram	Feature dimensions per block	Recognition rate (%)
Concatenating histograms	28	92.78
Stacking histograms	**14**	**94.44**

**Table 3:** Essential parameter settings for each descriptor.

Descriptor	Parameters	Value
LBP [17]	Block size	16 *∗* 16 pixels
	Bit numbers	8
CS-LBP [18]	Block size	16 *∗* 16 pixels
	Bit numbers	4
Gabor [19]	Direction numbers	8
	Scale numbers	5
	Sample interval	4
HOG [20]	Cell size	8 *∗* 8 pixels
	Block size	2 *∗* 2 cells
LDP [13]	Block size	16 *∗* 16 pixels
	Bit numbers	8
	Prominent direction numbers	3
LDNP [14]	Block size	16 *∗* 16 pixels
	Bit numbers	6
es-LBP [15]	Histogram categories	3
	Bin numbers per histogram	708/354/472
GDP [21]	Block size	16 *∗* 16 pixels
	Bit numbers	8
LDDSCP	Block size	16 *∗* 16 pixels
	Bit numbers	4

**Table 4:** The feature size and feature extraction time of different descriptors on the JAFFE database.

Descriptor	Feature size	Feature extraction time (s)
LBP [17]	16384	92.56
CS-LBP [18]	1024	6.18
Gabor [19]	40960	1332.15
HOG [20]	2304	**2.35**
LDP [13]	16384	86.33
LDNP [14]	4096	46.75
es-LBP [15]	1534	206.88
GDP [21]	16384	234.21
LDDSCP	**896**	7.84

**Table 5 tab5:** Facial expression recognition rates of different descriptors on two databases.

	LBP	CS-LBP	Gabor	HOG	LDP	LDNP	es-LBP	GDP	LDDSCP
JAFFE	86.67	88.89	95.56	90.56	89.44	88.33	87.22	83.89	94.44
CK	96.82	97.42	96.78	96.04	97.60	97.30	96.94	96.10	**97.96**

**Table 6:** Facial expression recognition rates of LDDSCP-1, LDDSCP-2, and LDDSCP on two databases.

	LDDSCP-1	LDDSCP-2	LDDSCP
JAFFE	93.33	92.22	**94.44**
CK	97.78	97.84	**97.96**

## Data Availability

The data used to support the findings of this study are included within the article.

## References

[1] Happy S. L., Routray A. (2015). Automatic facial expression recognition using features of salient facial patches. *IEEE Transactions on Affective Computing*.

[2] Eleftheriadis S., Rudovic O., Pantic M. (2015). Discriminative shared Gaussian processes for multiview and view-invariant facial expression recognition. *IEEE Transactions on Image Processing*.

[3] Torres B., Santos R. L., de Sousa M. F. B. (2015). Facial expression recognition in Alzheimer’s disease: a longitudinal study. *Arquivos de Neuro-Psiquiatria*.

[4] Dhall A., Ramana Murthy O. V., Goecke R., Joshi J., Gedeon T. Video and image based emotion recognition challenges in the wild: Emotiw 2015.

[5] Huang D., Shan C., Ardabilian M., Wang Y., Chen L. (2011). Local binary patterns and its application to facial image analysis: a survey. *IEEE Transactions on Systems, Man, and Cybernetics, Part C (Applications and Reviews)*.

[6] Jia S., Shen L., Li Q. (2015). Gabor feature-based collaborative representation for hyperspectral imagery classification. *IEEE Transactions on Geoscience and Remote Sensing*.

[7] Kyperountas M., Tefas A., Pitas I. (2010). Salient feature and reliable classifier selection for facial expression classification. *Pattern Recognition*.

[8] Li P., Phung S. L., Bouzerdom A., Tivive F. H. C. Improved facial expression recognition with trainable 2-D filters and support vector machines.

[9] Kim J., Yu S., Kim D., Toh K.-A., Lee S. (2017). An adaptive local binary pattern for 3D hand tracking. *Pattern Recognition*.

[10] Ryu J., Hong S., Yang H. S. (2015). Sorted consecutive local binary pattern for texture classification. *IEEE Transactions on Image Processing*.

[11] Tang Z., Su Y., Er M. J., Qi F., Zhang L., Zhou J. (2015). A local binary pattern based texture descriptors for classification of tea leaves. *Neurocomputing*.

[12] Davarzani R., Mozaffari S., Yaghmaie K. (2015). Scale-and rotation-invariant texture description with improved local binary pattern features. *Signal Processing*.

[13] Jabid T., Kabir M. H., Chae O. (2010). Robust facial expression recognition based on local directional pattern. *ETRI Journal*.

[14] Rivera A. R., Castillo J. R., Chae O. O. (2013). Local directional number pattern for face analysis: face and expression recognition. *IEEE Transactions on Image Processing*.

[15] Chao W.-L., Ding J.-J., Liu J.-Z. (2015). Facial expression recognition based on improved local binary pattern and class-regularized locality preserving projection. *Signal Processing*.

[16] Ding C., Choi J., Tao D., Davis L. S. (2016). Multi-directional multi-level dual-cross patterns for robust face recognition. *IEEE Transactions on Pattern Analysis and Machine Intelligence*.

[17] Ojala T., Pietikäinen M., Harwood D. (1996). A comparative study of texture measures with classification based on featured distributions. *Pattern Recognition*.

[18] Heikkilä M., Pietikäinen M., Schmid C. (2009). Description of interest regions with local binary patterns. *Pattern Recognition*.

[19] Ruan J. X. (2010). Study on key technology for multi-pose face detection and facial expression recognition.

[20] Dalal N., Triggs B. Histograms of oriented gradients for human detection.

[21] Ahmed F. (2012). Gradient directional pattern: a robust feature descriptor for facial expression recognition. *Electronics Letters*.

[22] Ojala T., Pietikainen M., Maenpaa T. (2002). Multiresolution gray-scale and rotation invariant texture classification with local binary patterns. *IEEE Transactions on Pattern Analysis and Machine Intelligence*.

[23] Huang D., Ardabilian M., Wang Y., Chen L. (2012). 3-D face recognition using eLBP-based facial description and local feature hybrid matching. *IEEE Transactions on Information Forensics and Security*.

[24] Huang D., Wang Y., Wang Y. A robust method for near infrared face recognition based on extended local binary pattern.

[25] Tan X., Triggs B. (2010). Enhanced local texture feature sets for face recognition under difficult lighting conditions. *IEEE Transactions on Image Processing*.

[26] Vu N. S., Caplier A. (2012). Enhanced patterns of oriented edge magnitudes for face recognition and image matching. *IEEE Transactions on Image Processing*.

[27] Wolf L., Hassner T., Taigman Y. (2011). Effective unconstrained face recognition by combining multiple descriptors and learned background statistics. *IEEE Transactions on Pattern Analysis and Machine Intelligence*.

[28] Wolf L., Hassner T., Taigman Y. Descriptor based methods in the wild.

[29] Kabir M. H., Jabid T., Chae O. A local directional pattern variance (LDPv) based face descriptor for human facial expression recognition.

[30] Luo Y.-T., Zhao L.-Y., Zhang B. (2016). Local line directional pattern for palmprint recognition. *Pattern Recognition*.

[31] Yuan F. (2014). Rotation and scale invariant local binary pattern based on high order directional derivatives for texture classification. *Digital Signal Processing*.

[32] Mu M., Ruan Q., Guo S. (2011). Shift and gray scale invariant features for palmprint identification using complex directional wavelet and local binary pattern. *Neurocomputing*.

[33] Caspers S., Axer M., Caspers J. (2015). Target sites for transcallosal fibers in human visual cortex–a combined diffusion and polarized light imaging study. *Cortex*.

[34] Stigliani A., Weiner K. S., Grill-Spector K. (2015). Temporal processing capacity in high-level visual cortex is domain specific. *Journal of Neuroscience*.

